# A Narrative Review of the High-Carbohydrate Fueling Revolution (≥ 100 g/h) in the Professional Peloton

**DOI:** 10.1007/s40279-025-02372-6

**Published:** 2025-12-04

**Authors:** Patrick B. Wilson

**Affiliations:** Human Performance Laboratory, School of Exercise Science, Ellmer College of Health Sciences, Old Dominion University, Norfolk, VA 23529 USA

## Abstract

High-carbohydrate fueling in cycling (defined as ≥ 100 g/h for this paper) has received significant media attention in recent years. Whether this practice improves performance, however, remains an unresolved issue in the scientific literature. The purpose of this narrative review is to provide an up-to-date analysis of the practice of high-carbohydrate fueling, with a specific focus on potential performance implications in professional cycling. Topics covered include historical carbohydrate intake guidelines, research directly comparing high-carbohydrate fueling with traditional fueling guidelines, theorized benefits of high-carbohydrate fueling specific to cycling, potential risks associated with high-carbohydrate fueling, and personalizing carbohydrate intakes. Among a small number of experimental studies that have compared high-carbohydrate fueling with somewhat lower rates (e.g., 60–90 g/h), there is not clear evidence that it reduces reliance on endogenous carbohydrate stores or improves performance. However, these studies have not closely mimicked the demands of multi-day and multi-week stage races, when ingesting carbohydrate at ≥ 100 g/h may be more likely to produce performance benefits. Observational data from professional cyclists suggest that carbohydrate consumption during racing is strongly associated with total daily carbohydrate intakes; therefore, ingesting carbohydrate at ≥ 100 g/h on the bike could facilitate performance over multiple days or weeks by enhancing glycogen resynthesis and recovery. In addition, circumstantial evidence suggests that high-carbohydrate fueling could reduce low energy availability, reduce within-day energy deficits, and stimulate the central nervous system. Personalizing carbohydrate intakes through individual assessments of exogenous carbohydrate oxidation is a novel strategy that should be further explored in the future.

## Key Points

**Table:** 

Existing research does not directly support performance-enhancing effects of ingesting carbohydrate at ≥ 100 g/h versus 60–90 g/h, but studies underpinning this conclusion do not closely reflect the conditions and demands that cyclists face when they participate in multi-day/multi-week stage races or intensified training periods.
Indirect evidence suggests that high-carbohydrate fueling could contribute to improved performance by enhancing daily carbohydrate availability, reducing low energy availability, reducing within-day energy deficits, and stimulating the nervous system via oral exposure.
Potential risks of high-carbohydrate fueling that should be weighed against possible benefits include suppressed fat oxidation, accelerated glycogen degradation, reduced metabolic/biochemical adaptations to training (if applied excessively), and gastrointestinal symptom exacerbation.

## Introduction

Multiple recent news articles and blogs have claimed that professional cycling and other endurance sports are in the midst of a ‘carbohydrate revolution’ [1–3]. One blog written by a professional ultra-runner and coach, for example, went as far as asserting that recent performance improvements across a range of endurance sports are primarily driven by nutrition, as the “only variable that connects all of the sports is simple: high-carbohydrate fueling” [3]. While the scientific merit of these sorts of claims is uncertain and contributions of other factors (technological advancements, equipment development, professionalization, etc.) should not be overlooked, the theoretical advantages of high-carbohydrate fueling (e.g., ≥ 100 g/h during exercise) have also been discussed in contemporary peer-reviewed articles [4, 5]. Clearly, there is growing interest in the practice of consuming carbohydrates at rates exceeding standard scientific guidelines (i.e., up to 90 g/h [6–8]).

**Fig. 1:**
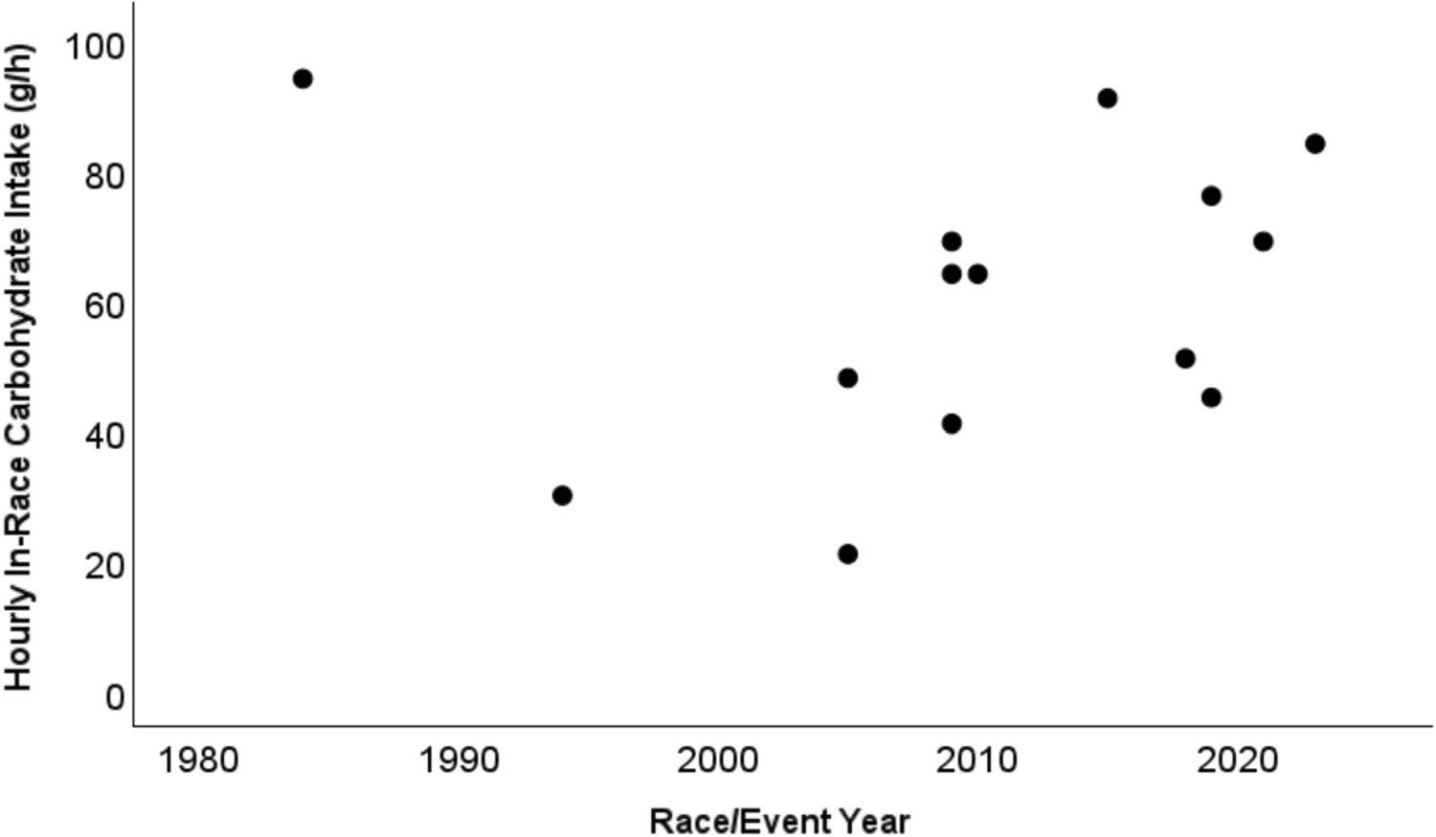
The relationship between event/race year and average hourly carbohydrate intakes from studies of professional road cycling races

Observational studies of elite/professional road cyclists provide some insight into how fueling practices may have changed over time [9–20]. Table 1 displays average hourly carbohydrate intakes from studies of road cycling events held between 1984 and 2023, and the relationship between event year and hourly intakes is presented in Fig. 1. The correlation between variables is insignificant when considering the entire sample (Pearson *r* = 0.12, *p* = 0.694). However, the investigation by Saris et al. of Tour de France riders from 1984 is a clear outlier [9], and a significant correlation exists between variables (*r* = 0.64, *p* = 0.019) after removing it from the analysis. Notably, Saris et al. [9] used rider diaries in combination with weekly checks by a nutritionist to evaluate intakes, which was cross-checked with information about food supplied during the race. Given that nutritionist checks occurred relatively infrequently and there was scarce detail on the method used to cross-check supplied versus consumed food, it is possible that carbohydrate intakes were overestimated. Regardless, whether any true temporal trends in carbohydrate intakes exist is difficult to quantify given the limited data as well as variations in specific races/events and riders studied. An examination of data from three Vuelta a Españas (1994, 2009, 2015) provides some additional support for the idea that in-race carbohydrate intakes may have increased over time (Table 1 [10, 13, 15]). Irrespective of the available data, there is a popular perception that carbohydrate ingestion during racing has increased in recent years [1, 2].

**Table 1:** Studies that have reported during-race carbohydrate intakes in professional / elite road cyclists

Study	Publication year	Sample	Race/event (number of stages assessed)^a^	During-race carbohydrate intake (g/h)
Saris et al. [9]	1989	5 males	1984 Tour de France (all stages^b^)	94
García-Rovés et al. [10]	1998	10 males	1994 Vuelta a España (3 stages)	30 ± 8^c^
Ebert et al. [11]	2007	8 males	2005 Tour Down Under (6 stages)	48 ± 15
Ebert et al. [11]	2007	6 females	2005 Tour De L’Aude Feminin (7 stages)	21 ± 7
Ross et al. [12]	2014	5 males	2009 Tour of Gippsland (5 road stages)	41 ± 24
Pfeiffer et al. [13]	2012	15 males	2009 Vuelta a España (3 stages) and 2009 Dauphine (2 stages)	64 ± 20
Sánchez-Muñoz et al. [14]	2016	6 males	2009 Tour of Andalusia (4 stages)	69 ± 23^d^
Ross et al. [12]	2014	5 males	2010 Tour of Geelong (2 road stages)	64 ± 24
Muros et al. [15]	2019	9 males	2015 Vuelta a España (all stages)	91 ± 15
Heikura et al. [16]	2019	6 males	2018 editions of Driedraagse de Panne, E3 Harelbeke, Gent-Wevelgem, Dvaars door Vlaanderen	51 ± 9
Scott et al. [17]	2020	6 males with type I diabetes	2019 Tour of California (7 stages)	76 ± 23
Pitt et al. [18]	2022	7 males with type I diabetes	2019 Tour of Slovenia (5 stages)	45 ± 16
Strobel et al. [19]	2022	1 male	2021 Vuelta a España (all stages)	69 ± 18
Areta et al. [20]	2024	1 female	2023 Tour de France Femmes (stages 1–7)	84 ± 13

Hourly intake rates were rounded to nearest whole number and presented as mean and, when available or calculable, standard deviation

^a^The event year was inferred for some studies by comparing stage distances reported in the paper against stage distances reported from www.procyclingstats.com

^b^1 cyclist only had data from weeks 2 and 3 of the race

^c^Value was estimated by dividing the reported per-stage absolute average intake (128 g) by average winning times for the three stages, which are available from www.procyclingstats.com/race/vuelta-a-espana/1994/

^d^Value was estimated by taking the reported per-stage absolute average intake (278 g) and dividing it by estimated finishing time (4:03 h:min), which was based on the average distance of four stages reported in the paper (161.9 km) and an average speed of 40 km/h, a typical speed in professional stage races

Considering the media and scientific interest in high-carbohydrate fueling, the aim of this paper is to review the science of ingesting carbohydrate at high rates during cycling (with a focus on professional road racing). The operational definition of high-carbohydrate fueling for this narrative review is at least 100 g/h. Topics covered include historical carbohydrate intake guidelines, research directly comparing high-carbohydrate fueling to amounts falling within traditional guidelines, theorized benefits and risks of high-carbohydrate fueling, and personalizing carbohydrate intakes. Additionally, gaps in existent literature and future directions are discussed.

## Historical Carbohydrate Guidelines

Research on carbohydrate ingestion during exercise goes back many decades [21], though the 1960s is when recognition of the importance of carbohydrate for performance crystalized. Swedish researchers at that time found strong relationships between muscle glycogen and exercise time to fatigue [22, 23]. The first widely available carbohydrate-based sports drink, Gatorade, was also developed in the 1960s and was influential in the emergence of sports nutrition products [24].

As research on carbohydrate supplementation progressed into the 1990s and 2000s, a common recommendation that emerged was to ingest 30–60 g/h [25]. The American College of Sports Medicine (ACSM) position stand on nutrition and performance, which was published in 2009, provides a prominent example near the end of this timeframe, stating that “during exercise, primary goals for nutrient consumption are to replace fluid losses and provide carbohydrates (approximately 30–60 g·h^−1^) for maintenance of blood glucose levels” [26].

In the 2010s, a series of papers laid out revised carbohydrate guidelines based on emerging science, with up to 90 g/h recommended for athletes partaking in prolonged exercise [6, 7]. In 2011, Burke et al. [7] wrote that, “new guidelines to promote individual experimentation with carbohydrate intakes of up to 90 g h^−1^ in ultraendurance sports are warranted….” Likewise, an updated ACSM position stand in 2016 [6] included a statement that “in very prolonged events (2.5 + h) or other scenarios where endogenous carbohydrate stores are substantially depleted, higher intakes (up to 90 g/h) are associated with better performance.” The increased recommended upper range (60–90 g/h) was primarily based on studies that showed when multiple transportable carbohydrate sources are ingested (i.e., maltodextrin/glucose + fructose), rates of exogenous carbohydrate oxidation can surpass 60 g/h [27, 28]. In fact, ingestion rates as high as 144 g/h, with an oxidation efficiency of over 70%, had been reported by 2008 [29]. In addition, an experiment by Smith et al. [30] published in 2013 found a curvilinear relationship between carbohydrate dose (from 0 to 120 g/h) and performance, and the estimated optimal consumption was 78 g/h, notably above the recommended intake ceiling of 60 g/h from earlier guidelines.

Although most guidelines around this time capped the upper end of carbohydrate ingestion at 90 g/h [6], some researchers were suggesting that even higher intakes could be worth trying. In 2014, for example, Stellingwerff and Cox [31] advised a range of intakes from 40 g/h to 110 g/h during exercise lasting over 2 h.

## Research Comparing High-Carbohydrate Fueling (≥ 100 g/h) with Traditional Carbohydrate Guidelines (30–90 g/h)

Although there are investigations going back to the 1980s and 1990s that fed carbohydrate at rates ≥ 100 g/h and compared them with lower doses [32, 33], this section primarily focuses on work published since the 2016 ACSM position stand. These contemporary studies tend to better reflect the nature of modern-day fueling practices (e.g., use of multiple transportable carbohydrates) or made sure to evaluate exogenous carbohydrate oxidation.

In an experiment published in 2018, King and colleagues [34] examined the impact of four different carbohydrate feeding rates on exogenous and endogenous carbohydrate use as well as performance in ten trained cyclists. Carbohydrate was supplied at 60, 75, 90, or 112.5 g/h during 2 h of cycling at 77% of *V*O_2max_, which was followed by a 30-min time trial. The two higher doses (90 and 112.5 g/h) supplied glucose–fructose (2:1 ratio) while the two lower doses provided glucose only. Interestingly, although both high-carbohydrate glucose–fructose conditions (90 and 112.5 g/h) increased exogenous carbohydrate oxidation relative to the glucose-only conditions (60 and 75 g/h), ingesting 112.5 g/h appeared to increase reliance on liver and muscle glycogen (moderate-to-large effects) relative to 90 g/h. In addition, the 112.5-g/h condition did not lead to higher exogenous carbohydrate oxidation than 90 g/h, and average power output during the time trial was highest with 90 g/h and ~ 5% higher than with 112.5 g/h, though this was statistically insignificant. The lack of a linear relationship between carbohydrate dose and performance is in line with observations from Smith et al. [30], as well as a 1989 experiment that fed carbohydrate at 0, 37, 74, and 111 g/h during 120 min of cycling [33]. In that study, the 74-g/h dose enhanced performance on a 15-min performance ride versus placebo, whereas the 111-g/h condition failed to improve performance against placebo [33].

A second study by King et al. [35] evaluated the effects of three different carbohydrate feeding rates (80, 90, and 100 g/h supplied as 2:1 glucose–fructose) and placebo during 3 h of cycling at 60% of *V*O_2max_. Performance was measured during a 30-min time trial following submaximal cycling. Exogenous carbohydrate oxidation during the last hour of submaximal exercise was approximately 10% higher with 100 g/h than with 80 and 90 g/h, while at the same time, the 100-g/h dose moderately increased muscle glycogen use and its relative contribution to energy expenditure versus 90 g/h. Additionally, performance on the 30-min trial was likely/probably improved with 90 g/h as compared to 80 and 100 g/h (power outputs: 80 g/h = 219 ± 32 W; 90 g/h = 228 ± 37 W; 100 g/h = 212 ± 48 W). Notably, most of the reported differences in exogenous and endogenous carbohydrate use were not statistically significant despite being moderately sized, and purported performance differences between conditions were based on a magnitude-based inferential analysis, an approach that has since been largely abandoned in sport science research [36]. The results should therefore be interpreted somewhat cautiously.

A subsequent investigation by Podlogar et al. [37] compared exogenous carbohydrate oxidation between 120 and 90 g/h provided in 1:0.8 or 2:1 maltodextrin-fructose ratios, respectively. Exercise consisted of 3 h of cycling at 95% of gas exchange threshold; no performance test was conducted. Overall, the authors reported ~ 17% higher exogenous carbohydrate oxidation from 120–180 min of exercise with 120 g/h versus 90 g/h (1.51 ± 0.22 g/min vs 1.29 ± 0.16 g/min; *p* = 0.026). In contrast to the King et al. [34, 35] studies, which found possible moderate-sized increases in endogenous carbohydrate use with the highest carbohydrate doses, Podlogar et al. [37] did not observe between-condition differences in endogenous carbohydrate use. A possible explanation for this is the makeup of the feedings. Glucose–fructose in a 2:1 ratio was used for the 90-g/h and 112.5-g/h conditions in King et al. [34] and for all three carbohydrate doses in King et al. [35], and there is some evidence that a ratio closer to 1:1 is optimal with rates > 90 g/h [5]. In addition, glucose may slow gastric emptying as compared to maltodextrin [38], which would potentially delay intestinal carbohydrate absorption and its delivery to muscle.

An important consideration for interpreting the experiments by King et al. [34, 35] and Podlogar et al. [37] is that endogenous glycogen use was deduced/estimated from tracer and whole-body substrate use data, meaning that glycogen was not measured with methods such as biopsies or magnetic resonance spectroscopy (MRS). Some, though not all, studies using biopsies or MRS have reported glycogen sparing with carbohydrate ingestion [39–41], albeit at lower feeding rates than the highest doses used in King et al. [34, 35] and Podlogar et al. [37]. Consequently, future experiments may consider using more direct measurements of muscle glycogen to confirm or refute the purported increased endogenous use with high-carbohydrate fueling.

In summary, findings from Podlogar et al. [37] suggest that when carbohydrate is ingested at 120 g/h (1:0.8 maltodextrin–fructose ratio), exogenous carbohydrate oxidation can be elevated beyond rates achieved with 90 g/h of carbohydrate (2:1 maltodextrin–fructose ratio). However, when a glucose–fructose ratio of 2:1 is used, an increase in exogenous carbohydrate oxidation may not occur when carbohydrate is fed at > 110 g/h. Furthermore, the lack of endogenous carbohydrate sparing in Podlogar et al. [37], along with the possible increased reliance on endogenous carbohydrate and failure to improve performance with the highest carbohydrate doses in King et al. [34, 35], raise questions as to whether intakes above 100 g/h have any positive impacts on real-world cycling performance. This skepticism is also re-enforced by Smith et al. [30] and Mitchell et al. [33], who failed to find performance advantages with the highest carbohydrate feeding rates versus somewhat lower doses.

Even if current literature does not directly support performance advantages of high-carbohydrate fueling, there has been suggestion that this practice facilitates quicker muscle and functional recovery [3]. This speculation largely comes from an experiment by Viribay et al. [42], who conducted a randomized parallel-group trial with 20 runners participating in a mountain marathon. Runners were randomized to consume 60, 90, or 120 g/h of carbohydrate in the form of 30-g maltodextrin–fructose (2:1 ratio) gels during the race. Several blood markers of exercise-induced muscle damage were measured pre- and post-race, including creatine kinase (CK), lactate dehydrogenase (LDH), glutamic oxaloacetic transaminase (GOT), urea, and creatinine. Of these markers, increases from pre-race to post-race for CK, LDH, and GOT were suppressed in the 120-g/h group relative to the other groups. In a second paper, the authors reported that the 120-g/h group experienced better recovery on several performance tests (jumping, half-squatting, high-intensity running capacity time) [43].

While these results suggest that ingesting carbohydrate above 100 g/h during muscle-damaging exercise may enhance recovery, a cautious interpretation of the results is warranted. Sample sizes for each group were only 6–7 participants, which is understandable given the limited availability of highly trained and elite athletes in this setting, but it also means the chances of non-replicability and effect-size inflation are higher [44]. In addition, we might expect there to be a dose–response relationship, with 90 g/h having better outcomes than 60 g/h, and 120 g/h having better outcomes than 90 g/h. However, no consistent improvements were observed with 90 g/h versus 60 g/h. The mechanism by which increasing carbohydrate from 90 to 120 g/h would improve markers of muscle damage, with an absence of improvement when going from 60 to 90 g/h, is unclear. In addition, applicability of these findings to cyclists is unknown given that muscle damage markers typically show smaller increases with cycling than mountain marathoning [42, 45].

## Theoretical Benefits of High-Carbohydrate Fueling

The findings of several laboratory experiments do not support the idea that carbohydrate ingested at ≥ 100 g/h increases performance relative to 60–90 g/h [30, 33–35]. However, the absence of evidence does not necessarily mean that high-carbohydrate fueling truly fails to influence performance outside of the laboratory. Anecdotally, many professional cyclists believe in the importance of high-carbohydrate fueling, and accounts of its positive impact exist at the researcher–practitioner interface. James Morton, a researcher who worked with Team Sky (now known as INEOS Grenadiers), has written about the possible role of high-carbohydrate fueling in Chris Froome’s remarkable stage 19 performance in the 2018 Giro d’Italia [46]. Froome consumed nearly 600 g of carbohydrate (estimated ~ 100–110 g/h) on the bike during that stage, which culminated in a long-range attack on the Colle delle Finestre.

There are several theoretical benefits to high-carbohydrate fueling, which, albeit speculative, are worth discussing. Possible advantages include enhancing overall daily carbohydrate availability, reducing risks of low energy availability, reducing within-day energy deficits, stimulating the central nervous system, and possible tastant and placebo effects.

### Increased Total Carbohydrate Intake

Cyclists regularly ingesting high amounts of carbohydrate on the bike may, regardless of intention, end up consuming higher daily amounts of carbohydrate, which could translate to improved energy balance, glycogen levels, and possibly neuroendocrine and hormonal function [47]. These theoretical benefits are probably most relevant during multi-week stage races (e.g., Grand Tours), when performance is dependent on large cumulative carbohydrate intakes. A recent experiment by Fuchs et al. [48] highlights the potential importance of achieving high daily carbohydrate intakes in cyclists when turnaround time between stages is limited. Specifically, aggressive carbohydrate refueling with primarily sucrose (10 g/kg of body mass) after exhaustive exercise failed to fully restore muscle glycogen within 12 h (glycogen reached 69% of pre-exercise levels). It is possible that ingesting carbohydrate at high rates on the bike, rather than waiting to prioritize refueling post-exercise, may speed up this process of glycogen recovery. Limited research has shown that glycogen can be synthesized during light or mild exercise, particularly in type II muscle fibers [49, 50], but whether this occurs in cyclists during competition characterized by higher relative workloads is more doubtful. In addition, Zachwieja et al. [51] reported that, in comparison with placebo, carbohydrate ingestion (~ 86 g/h) during 2 h of cycling at 70% of *V*O_2max_ led to no further enhancement of post-exercise glycogen synthesis. Despite the equivocal evidence, it is reasonable to suggest that any ingested carbohydrate that goes unoxidized, especially if consumed near the end of exercise, could contribute to muscle glycogen resynthesis post-exercise. While this may be relatively inconsequential for recovery from a single bout of exercise, cumulative effects over multiple weeks could be more substantial.

**Fig. 2:**
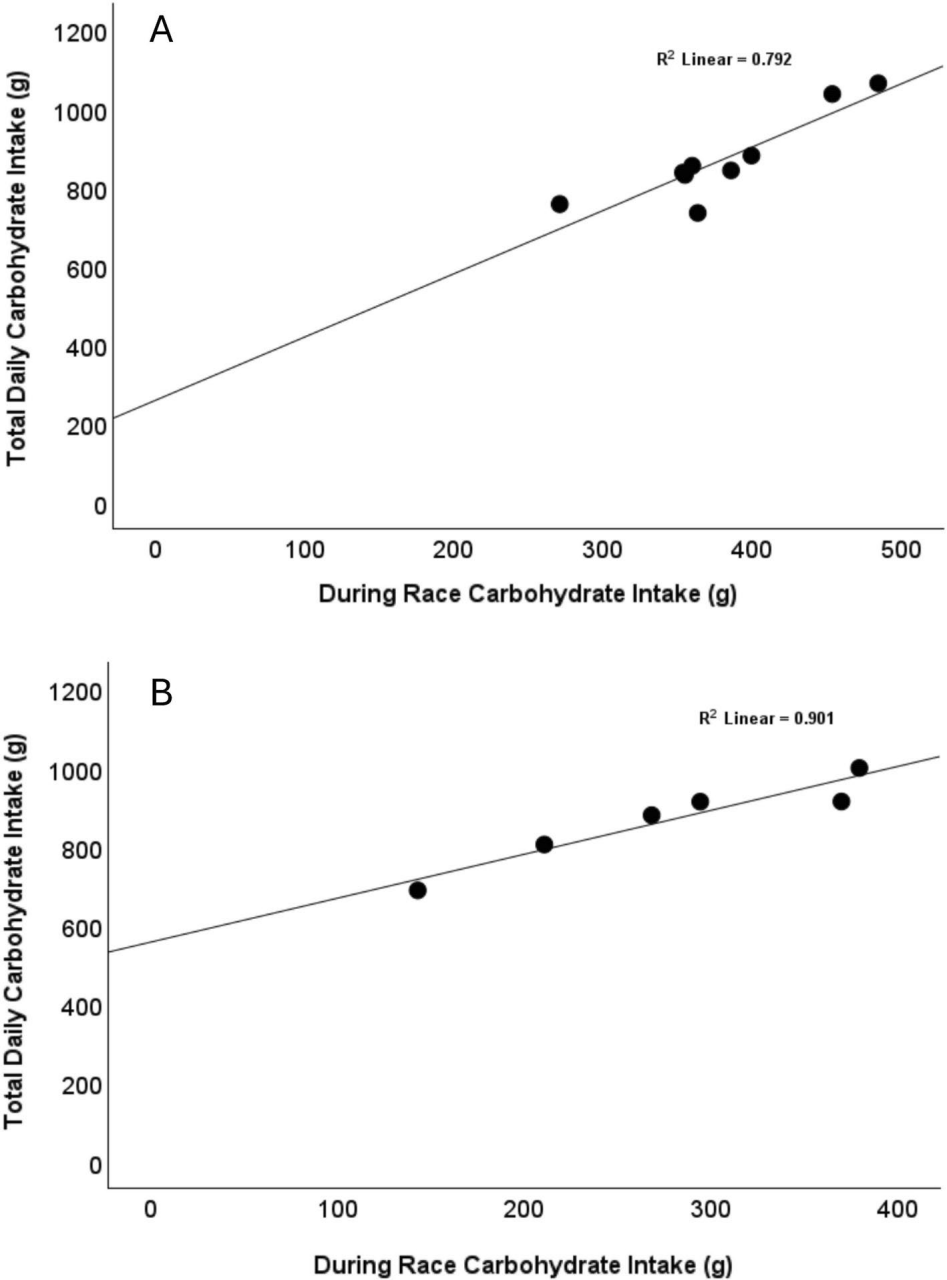
Association between during-race carbohydrate intake and total daily carbohydrate intake based on data from Muros et al. [15] (panel **A**) and Sánchez‐Muñoz et al. [14] (panel **B**)

**Fig. 3:**
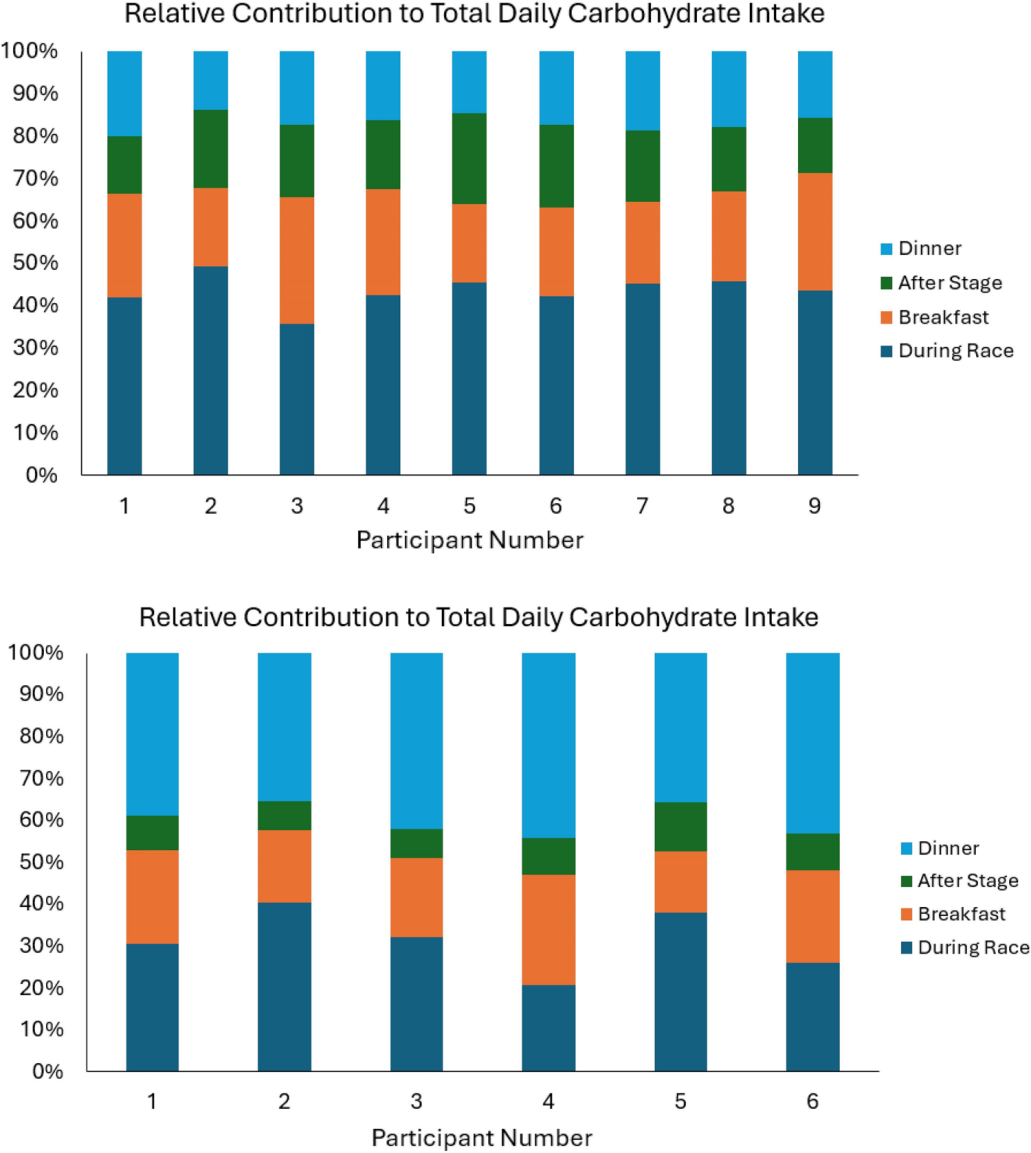
Relative contribution of different meals/feedings to total daily carbohydrate intakes from Muros et al. [15] (top panel) and Sánchez‐Muñoz et al. [14] (bottom panel)

The following scenario illustrates how altering carbohydrate ingestion practices on the bike could contribute to larger total carbohydrate intakes over the duration of a multi-week stage race. A cyclist consuming an average of 70 g/h during the Tour de France would ingest approximately 5600 g of carbohydrate during exercise over the event (assuming a completion time of 80 h). By increasing this to 110 g/h, total carbohydrate consumption during racing would increase to 8800 g, providing an extra 3200 g of total carbohydrate if consumption at non-exercise feedings/meals remains stable. While a cyclist ingesting 110 g/h on the bike might down-regulate carbohydrate intake at non-exercise feedings/meals and thereby lessen the difference in total intake over the event, data from a pair of observational studies of professional male cyclists suggest that on-bike carbohydrate fueling is a driver of total daily carbohydrate intake [14, 15]. Each study used a combination of food weighing and self-report to assess dietary intake on race days, with reporting of carbohydrate intakes for individual cyclists at several feedings/meals (breakfast, during racing, after racing, dinner). Pearson correlations between carbohydrate intakes at each feeding/meal and total daily carbohydrate intake, which are based on data extracted from the papers, are shown in Table 2. Carbohydrate intakes during racing were very strongly correlated with total daily carbohydrate intakes in both samples (*r* ≥ 0.89, *p* < 0.01), while none of the other variables showed statistically significant associations. A visual representation of the associations between carbohydrate ingestion during racing and total daily carbohydrate is shown in Fig. 2. In addition, Fig. 3 displays the relative contribution of each feeding/meal to total daily carbohydrate intake. Notably, carbohydrate during racing was the largest contributor to total daily carbohydrate intake for all nine cyclists in Muros et al. [15], which was conducted at the 2015 Vuelta a España.

**Table 2:** Correlations between carbohydrate intakes from different meals/feedings and total daily carbohydrate intakes

	Muros et al. [15] (*n* = 9)	Sánchez-Muñoz et al. [14] (*n* = 6)
	Total daily CHO	Total daily CHO
During race CHO	0.89 (0.001)	0.95 (0.004)
Breakfast CHO	0.54 (0.131)	− 0.57 (0.240)
After race CHO	0.60 (0.090)	0.65 (0.167)
Dinner CHO	0.65 (0.060)	0.66 (0.151)

Values shown are *r* (*p* value)

*CHO* carbohydrate

While these analyses support the hypothesis that during-race carbohydrate intake is an important determinant of total daily carbohydrate intake, they should be interpreted cautiously given the small sample sizes and that the dependent variable (total daily intake) includes the predictor variable (during-race intake), meaning the two variables are not fully independent. Ultimately, it would be valuable for future experimental studies to evaluate how increasing carbohydrate ingestion while cycling affects carbohydrate intake at other feedings and any associated changes in daily carbohydrate totals.

### Avoiding Low Energy Availability

Low energy availability, which is characterized by a lack of energy intake needed to sustain physiological functions outside of exercise [52], affects a substantial number of endurance athletes, including professional cyclists [53]. Low energy availability is proposed to be the main underlying cause of relative energy deficit in sport (REDs), a syndrome of negative physiological, health, and performance effects [54]. It has recently been argued that problematic low energy availability, a severe maladaptive version, is the main concern and is associated with potentially persistent disturbances in multiple body systems [55]. Adaptative low energy availability, in contrast, is thought to have mostly benign consequences, “including mild and quickly reversible changes in biomarkers of various body systems that signal an adaptive partitioning of energy and the plasticity of human physiology” [55].

As discussed in Sect. 4.1, cyclists consuming more carbohydrate on the bike may increase total carbohydrate consumption throughout a Grand Tour. Even a relatively modest increase in on-bike fueling during a Grand Tour, such as an extra 20–25 g/h, could raise energy intake (~ 300–400 kcal/day) and energy availability. Similar daily increases have been tied to restoration of menses in amenorrheic female athletes [56], bolstering the argument that even modest increases in energy intake, if sustained over time, could confer benefits.

Potential improvements in energy availability from on-bike high-carbohydrate fueling is at least partly supported by individual-level data from Sánchez‐Muñoz et al. [14] and Muros et al. [15]. Energy availability in these studies can be reasonably approximated by taking the energy intake reported for each rider, subtracting estimated exercise energy expenditure (calculated by assuming an energy cost of 0.32 kcal per kg of body mass per km), and dividing the result by fat-free mass (i.e., individual body masses multiplied by average pre-race fractional percentage fat-free mass from the Durnin and Womersley prediction reported in the papers). The estimated energy cost of 0.32 kcal per kg of body mass per km was derived by dividing during-stage energy expenditure estimates (from power data in Muros et al. [15]) by body mass, and then dividing the result by average stage distance; the obtained value is similar to 0.36 reported from a sample of recreational cyclists competing in a 4-h race [57]. Estimated mean (minimum, maximum) energy availabilities were 34.9 (19.0, 43.2) and 28.8 (20.1, 44.2) kcal per kg of fat-free mass in Sánchez‐Muñoz et al. [14] and Muros et al. [15], respectively. Pearson correlations between during-race carbohydrate intake and energy availability range from *r* = 0.54 (*p* = 0.130) in Muros et al. [15] to *r* = 0.86 (*p* = 0.029) in Sánchez‐Muñoz et al. [14], suggesting moderate-to-very-strong associations exist between these variables in the context of multi-day/week competition.

In addition to potentially improving energy availability during multiple days or weeks of competition, increased on-bike fueling could also reduce the incidence of low energy availability on specific days, especially days with high energetic demands. Taylor et al. [58] evaluated energy availability in 10 elite male road cyclists during the late pre-season and found substantial day-to-day fluctuations (range: − 22 to 76 kcal per kg fat-free mass per day). A separate study with six professional male cyclists examined energy availability during an 8-day window of racing (four single-day races) during the 2018 Spring Classics [16]. The authors reported that cyclists with extremely low energy availability on race days (< 10 kcal per kg fat free mass) showed a trend toward decreased testosterone (− 14%) and insulin-like growth factor-1 (− 25%), even though energy availability was normal on rest days. However, among the entire sample, there were no significant changes in body composition or several key hormones (testosterone, insulin-like growth factor-1, or cortisol) over the 8 days, which suggests that a rolling average may take precedence over individual daily variations in energy availability, at least in men [59].

Beyond contributing to low energy availability, low carbohydrate intakes may also have energy-independent effects on several aspects of REDs. As reviewed in the 2023 International Olympic Committee consensus statement on the topic, many of the studies that demonstrate negative effects of low energy availability are potentially confounded by low carbohydrate availability [54]. Indeed, the authors wrote that between the release of the 2018 and 2023 consensus statements, six studies demonstrated energy-independent and/or magnifying effects of low-carbohydrate availability on various REDs outcomes [54].

Though speculative, the totality of evidence suggests that on-bike carbohydrate intake could modify the risk of low energy availability during multi-week competitions or during intensified periods of the cycling season. Future studies should investigate whether interventions aimed at increasing carbohydrate fueling during exercise improve overall energy intake in athletes with low energy availability. This may be particularly relevant for cycling given the large amount of daily training time cyclists undertake relative to athletes in other sports like distance running [60, 61]. In addition, further research is needed to better understand the interactions between low energy availability and the energy-independent effects of carbohydrate in professional cycling.

### Better Within-Day Energy Matching

Cyclists, even if they have adequate daily energy availability, could still experience relatively large within-day energy deficits. Within-day energy balance refers to energy balance as the day progresses and is calculated based on hour-by-hour energy intakes and energy expenditures [62]. As an extreme example, an athlete who eats little-to-nothing from the time they wake (7 a.m.) until late afternoon could have a neutral daily energy balance if they eat enough energy at dinner and before going to sleep. However, they would be spending most of the day (on an hour-by-hour basis) in a negative energy balance and catabolic state.

The concept of within-day energy balance has been applied to endurance athletes in a limited number of studies. Among 31 male runners, cyclists, and triathletes, participants with suppressed resting metabolic rates (defined as having a ratio of measured/predicted of < 0.9) spent more time in an energy deficit exceeding 400 kcal and had larger single-hour energy deficits than athletes with normal resting metabolisms [63]. Moreover, having larger single-hour energy deficits was moderately associated with higher cortisol and a reduced testosterone:cortisol ratio. A similar study with 25 female endurance athletes found that those with menstrual dysfunction spent more time in a negative energy balance than eumenorrheic athletes, even though 24-h energy availability was not significantly different between the groups [64]. In addition, more hours with an energy balance of < 0 kcal and < − 300 kcal was moderately associated with suppressed metabolic rate, lower estradiol, and higher cortisol.

**Fig. 4:**
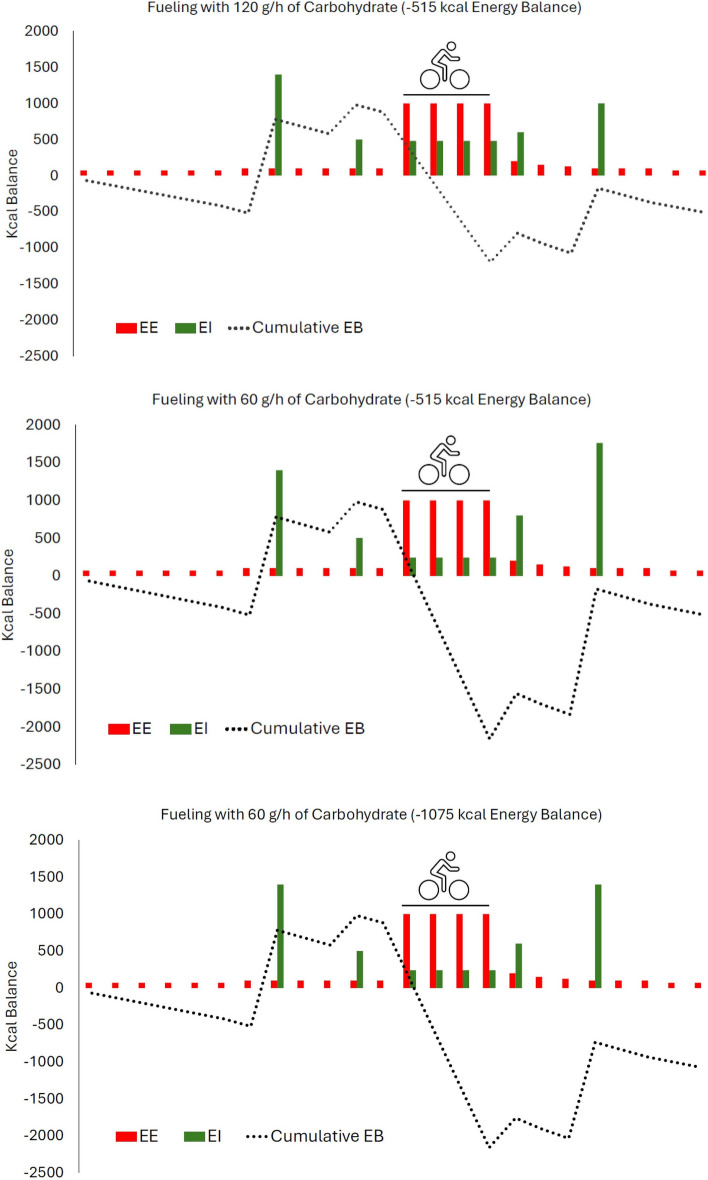
Within-day energy balance across 24 h under three scenarios. Scenario 1 (top panel): a cyclist consuming 120 g/h of carbohydrate during 4 h of racing. Scenario 2 (middle panel): a cyclist consuming 60 g/h of carbohydrate during 4 h of racing, while also increasing carbohydrate intake post-race to compensate for a lower in-race intake. Scenario 3 (bottom panel): A cyclist consuming 60 g/h of carbohydrate during 4 h of racing but who fails to adequately increase carbohydrate post-race. *EB* energy balance, *EE* energy expenditure, *EI* energy intake

Within-day energy balance may be particularly relevant in cycling, as athletes routinely spend 3–6 h on the bike per day for multiple days or weeks during competitions. The substantial energy expenditures during stages (up to 1000 kcal per hour) may predispose cyclists to greater time spent with large hourly deficits, especially when during-exercise carbohydrate intakes are low [65]. This concept is illustrated in Fig. 4, which shows within-day energy balance under three theoretical race scenarios. The first is a cyclist eating 120 g/h of carbohydrate during 4 h of racing, the second is a cyclist eating 60 g/h during racing who also increases carbohydrate intake post-race to compensate for a lower in-race intake, and the third is a cyclist ingesting 60 g/h but failing to adequately increase carbohydrate post-race. In the first two scenarios, overall energy balance is the same at the end of the day (− 515 kcal), but cyclist 2 has a more extreme negative energy balance for several hours after the race than cyclist 1. The third cyclist not only has a larger energy deficit at the end of the day (− 1075 kcal), but has also spent more time with a substantial energy deficit than cyclists 1 and 2. Summing the hourly cumulative energy balances also demonstrates that there are substantial differences between the scenarios (scenario 1 = − 4380; scenario 2 = − 9060; scenario 3 = − 12,460).

**Fig. 5:**
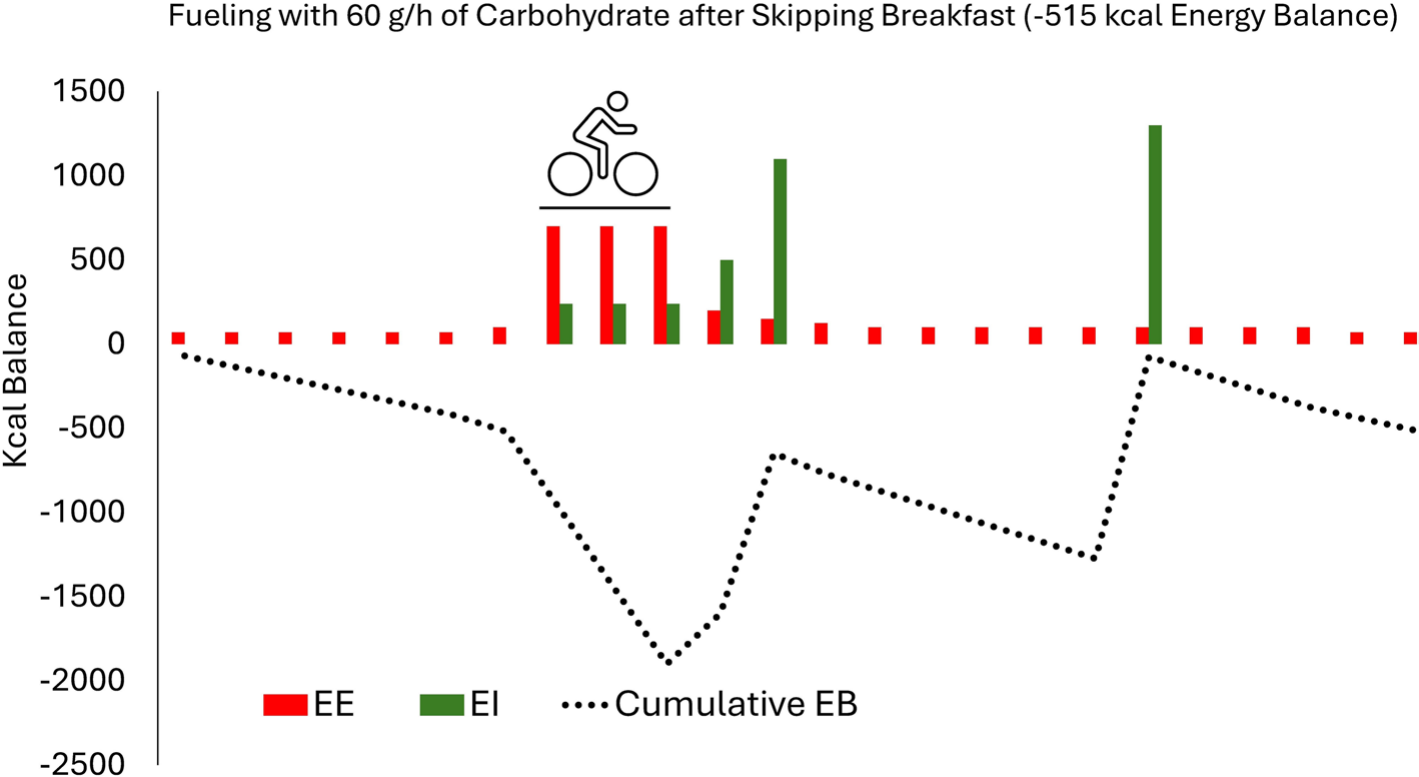
Within-day energy balance across 24 h for a cyclist who trains in the morning after skipping breakfast and consumes 60 g/h during a 3-h training ride. *EB* energy balance, *EE* energy expenditure, *EI* energy intake

Beyond racing situations, certain training practices, such as conducting sessions in the morning after skipping breakfast, can further exacerbate these within-day energy imbalances. While a professional cyclist is unlikely to skip breakfast before a road race, notable percentages of elite/professional endurance athletes do report at least occasionally engaging in fasted training [66]. Figure 5 shows a theoretical scenario for a cyclist who skips breakfast and chooses to fuel with 60 g/h of carbohydrate during a 3-h morning training session. Despite the lower exercise energy expenditure as compared with the three racing scenarios, the sum of hourly cumulative energy balances is worse (− 16,570).

Notably, existing research on within-day energy balance has primarily relied on cross-sectional designs without the assessment of REDs. While it seems obvious that low energy availability and poor within-day energy balance are likely to go hand in hand in many cases, there is only limited research to directly support this in cyclists. One small study of competitive female cyclists (*n* = 8) did find strong correlations between time with an energy balance under − 300 kcal and energy availability (*r* = − 0.74, *p* = 0.037) [67], but it remains unclear how low energy availability and within-day energy balance interact with one another over time. Although it will be challenging, future studies should consider employing prospective designs while assessing energy availability and within-day energy balance to better understand how the interplay of these two phenomena affect athlete health and performance.

### Central Nervous System, Tastant, and Placebo/Expectancy Effects

The main physiological benefits of carbohydrate consumption during exercise are thought to be sparing muscle glycogen, maintaining or increasing carbohydrate oxidation, and preventing hypoglycemia [68]. Research beginning in the 2000s, however, showed that exposing the mouth to carbohydrate beverages, even in the absence of swallowing, sometimes enhances performance in tasks lasting 30–60 min [69, 70]. These positive effects of carbohydrate mouth rinsing are amplified in situations when athletes are fasted or in a glycogen-depleted state [71]. Whether this translates to any performance benefits under conditions that match the demands of elite road cycling (races lasting 3–6 h with relatively high carbohydrate availability before exercise) has not been directly tested. Even so, a review of pertinent literature on carbohydrate mouth rinsing is justified given that this could be an area for future research.

While the applicability of exercise lasting 30–60 min to elite road cycling is likely minimal, several studies have examined the effects of carbohydrate rinsing at the end of longer-duration exercise or after glycogen depletion. Luden et al. [72] had trained cyclists complete approximately 3 h of cycling (2 h at 55% of watt_max_ plus a 30-km time trial), which was then followed by isometric force testing, 10 min of constant load cycling, and a 2-km time trial. Water was provided during the initial 3 h of cycling while three 5-s carbohydrate, protein, or placebo mouth rinses were administered in a crossover fashion during the isometric force testing and 10-min constant load cycling. The study revealed that carbohydrate rinsing ‘likely’ enhanced 2-km performance versus placebo (by 3.8%) based on magnitude-based inferences, though the *p* value was 0.11.

Jensen et al. [73] assigned 10 male cyclists to two experimental trials in a crossover fashion, each consisting of a standardized pre-trial snack (2 h prior) followed by 120 min of cycling at 60% *V*O_2max_ and then a time trial lasting approximately 30 min. The two experimental conditions were as follows: (1) 30 g/h of carbohydrate ingestion during the 120-min bout plus carbohydrate mouth rinsing during the time trial, and (2) 30 g/h of carbohydrate ingestion during the 120-min bout plus placebo mouth rinsing during the time trial. Overall, there was a 1.7% improvement in time trial time with carbohydrate rinsing versus placebo rinsing. Based on magnitude-based inferential statistics, this difference had a 60% probability of being a positive effect, though the *p* value was 0.51.

While Luden et al. [72] and Jensen et al. [73] found small possible (though non-statistically significant) benefits of carbohydrate rinsing under conditions that somewhat mimic the duration of some road cycling events, other similar investigations have failed to find any benefits whatsoever. Ali et al. [74] compared mouth rinsing a 15%-carbohydrate beverage, ingesting a 7.5%-carbohydrate beverage, mouth rinsing a placebo, and ingesting a placebo in a crossover experiment. Participants underwent 90 min of glycogen-reducing exercise and ate a low-carbohydrate post-exercise meal in the evening; the following morning, participants performed a fasted 1-h cycling time trial while treatments were administered eight times at even intervals. Under low-carbohydrate availability, power output was higher with carbohydrate ingestion relative to the other trials (*p* < 0.01), while carbohydrate rinsing failed to outperform placebo rinsing and placebo ingestion.

In sum, weak evidence indicates that oral exposure to carbohydrate could confer small positive effects to endurance cycling in some circumstances (fasted or glycogen-depleted states). In the context of the high-carbohydrate fueling practices of cyclists, an ingestion rate ≥ 100 g/h could lead to either more frequent or more intense carbohydrate exposure in the oral cavity as compared with lower feeding rates. Clearly, additional research is needed to examine whether the increased frequency or intensity/concentration of feeding associated with high-carbohydrate fueling is a factor in modifying performance. Isolating these effects from the metabolic effects of carbohydrate delivery to muscle will be challenging but could be facilitated by mechanistic work examining brain activation patterns and other neural-based assessments [75, 76].

Tastant effects from other ingredients and substances added to carbohydrate supplements could also hypothetically work through non-metabolic pathways to contribute to performance effects from high-carbohydrate fueling. As reviewed by Best et al. [77], rinsing bitter (quinine, caffeine) and thermal (menthol) tastants has elicited performance effects in limited circumstances, though most of the available data are not based on exercise tasks that mimic road cycling. Nonetheless, given the hundreds of unique carbohydrate products (gels, beverages, chews, etc.) on the market, many of which contain caffeine and flavor enhancers, the potential impact of different tastants should be considered in future research. By practicing high-carbohydrate fueling, cyclists are not only consuming greater amounts of carbohydrate per hour but are also possibly increasing their exposure to other tastants through more frequent feedings.

A final conjectural benefit of high-carbohydrate fueling is placebo/expectancy effects. Both medical and sport science research highlight the positive influence that placebos—and related interventions like positively framed written, oral, or visual information—can have on outcomes like pain, perceived effort, and performance [78, 79]. These positive effects may even occur in cases where individuals are fully aware they are receiving a placebo (i.e., ‘open-label placebo’) [80]. Given the anecdotal popularity of high-carbohydrate fueling within the professional peloton [1, 2], it could be speculated that more frequent feeding occasions during racing could act as a form of placebo to induce transient performance effects. This is analogous to observations that multiple pills are perceived as more effective than one pill, larger pills are perceived as more potent than smaller pills, and multiple doses are perceived as more potent than fewer doses [81].

## Potential Risks of High-Carbohydrate Fueling

The purported benefits of high-carbohydrate fueling are usually the focus of lay articles [1–3], often without adequate discussion of potential risks and unintended consequences. As with all interventions, there are advantages, disadvantages, and tradeoffs to be considered with high-carbohydrate fueling. Possible disadvantages include suppression of fat oxidation, acceleration of glycogen degradation, an increase in gastrointestinal symptoms, and reduced adaptations to training.

### Suppressed Fat Oxidation

Supplying exogenous carbohydrate before and/or during exercise is recognized as an important determinant of performance in high-level endurance activities like cycling [6, 7], and most studies that have fed carbohydrate during exercise lasting over 1 h have found improved performance [31]. However, a greater capacity to oxidize fat has been associated with better performance in a number of endurance athlete populations [82–84], implying that a cyclist’s ability to oxidize fat could be an important performance factor in certain situations, particularly ultra-endurance competitions. In addition, others have argued that maintaining fat oxidation, in part by limiting the amount of ingested carbohydrate, is a strategy worth considering in some endurance exercise contexts [85].

As mentioned, most studies have found that carbohydrate ingestion during endurance exercise improves performance [31]. However, in the small number of studies that have supplied ≥ 100 g/h against somewhat lower doses, these high-carbohydrate doses have typically failed to further improve performance, and in some cases performance appeared to worsen [30, 33–35]. While other mechanisms may account for these observations, it is impossible to completely rule out a role for suppressed fat oxidation. For example, there could be a tipping point that occurs at carbohydrate doses ≥ 100 g/h, where reduced fat oxidation becomes more problematic, especially if coupled with accelerated breakdown of muscle glycogen [85]. Available evidence generally supports the notion that increasing exogenous carbohydrate ingestion during exercise reduces fat oxidation in a dose-dependent manner, though this relationship may not be completely linear [34, 86, 87].

Whether reduced fat oxidation from high-carbohydrate fueling translates to any impairments in performance within the context of professional cycling is presently unclear. Studies connecting fat oxidation to performance have generally evaluated peak rates under fasted conditions and/or did not feed carbohydrate at high rates during performance testing [82–84]. Moreover, whole-body substrate utilization may simply be correlated to (but not causally related to) performance, so both existing and future data need to be interpreted carefully. Ultimately, it may be that metabolic flexibility—an ability to utilize endogenous fat at high rates while also being able to oxidize high rates of exogenous carbohydrate—is a more important determinant of success in disciplines like road cycling [88], though this requires further study.

### Accelerated Glycogen Degradation

As was discussed in Sect. 3, ingesting carbohydrate at or above 100 g/h could lead to slightly higher reliance on muscle glycogen. King et al. [34] reported that glucose–fructose fed at 90 and 112.5 g/h increased exogenous carbohydrate oxidation versus 60 and 75 g/h of glucose only, but they also found that 112.5 g/h increased reliance on endogenous carbohydrate stores in comparison with 90 g/h. In a separate study comparing 80, 90, and 100 g/h of glucose–fructose (2:1 ratio) fed during 3 h of exercise at 60% of *V*O_2max_, the 100-g/h condition likely increased muscle glycogen use during the final hour of exercise (*d* = 0.68), as well as its relative contribution to energy expenditure (*d* = 0.72), as compared with 90 g/h [35].

The performance implications of these possible modest differences in muscle glycogen utilization are unknown. King et al. [35] did report that performance on a 30-min performance trial was likely/probably (86.5–93% chance) improved with 90 g/h compared with 80 and 100 g/h. However, the underlying mechanism for why performance would be negatively impacted at feeding rates both slightly above and slightly below 90 g/h is not obvious. In addition, even if future studies confirm that increasing carbohydrate ingestion to ≥ 100 g/h impairs performance, it does not necessarily mean that a greater reliance on endogenous stores is the cause of such performance changes. Other possible explanations to consider include gastrointestinal discomfort, reduced fat oxidation, and hormonal changes. Furthermore, given some of the assumptions involved with estimating glycogen use via tracer-based methods, it would be valuable for future studies to consider using biopsies or MRS to assess glycogen levels.

### Gastrointestinal Disturbances

A variety of observational and experimental studies have shown that carbohydrate dose is related to the risk/severity of gastrointestinal symptoms, some of which are severe enough to impact performance [13, 89–91]. Unfortunately, gastrointestinal symptoms were not assessed in Smith et al. [30] and King et al. [34, 35], which failed to find enhanced performance with the highest carbohydrate doses (i.e., ≥ 100 g/h). Mitchell et al. [33, 92], who also failed to find a linear dose–response association between carbohydrate feeding and performance, reported that gastric emptying was lowest and gastric secretion was highest in the 111-g/h condition, though subjective ratings of stomach fullness did not differ between doses [92]. Though speculative, the absence of a linear relationship between carbohydrate dose and performance that has been observed in these studies [30, 33–35] could be due to increased gastrointestinal intolerance at the highest feeding rates.

The use of multiple transportable carbohydrates represents one approach to mitigate the gastrointestinal side effects associated with ingesting carbohydrate at high rates [27, 28], yet this does not eliminate symptoms in all athletes [93, 94]. Likewise, a systematic review [89] reported that exposing the gastrointestinal tract to relatively high rates of carbohydrate during training over 2 weeks can reduce gastrointestinal symptoms and carbohydrate malabsorption by as much as half, though a proportion of athletes still experience notable levels of symptoms post-gut training [95]. Regarding the aforementioned experiments that failed to observe performance improvements with carbohydrate doses ≥ 100 g/h versus somewhat lower doses, it is unknown whether participants had much, if any, prior experience with ingesting such high rates of carbohydrate. Thus, the possibility exists that performance could have been improved with doses ≥ 100 g/h if the athletes had been sufficiently familiarized with such high intake rates.

There is individuality to not only the severity of gastrointestinal symptoms a cyclist will experience to a given carbohydrate ingestion rate, but also to how well they will respond to a carbohydrate-focused gut training protocol [89, 95]. While reductions in carbohydrate malabsorption and gastrointestinal symptoms with gut training are documented [89, 95], any downstream changes to exogenous carbohydrate oxidation capacity are only supported by indirect evidence [96]. Interested readers are referred to a recent paper within a Union Cycliste Internationale (UCI) series that includes a discussion of gut training in elite cycling [97].

### Reduced Adaptation to Training

Investigations spanning the past 25 years furnish evidence that altering carbohydrate availability before, during, and after exercise can alter multiple biochemical and functional adaptations to training, including mitochondrial enzyme activities, mitochondrial content, and lipid oxidation capacity [98]. While differences in biochemical, hormonal, and enzymatic adaptations have frequently been observed in experiments that manipulate carbohydrate availability around exercise, alterations to performance have been less consistent [98, 99]. Nonetheless, there are examples from the literature of improved performance from conducting some training sessions with reduced carbohydrate availability. In one example, Marquet et al. [100] divided 21 triathletes into two groups, consisting of either sleeping low (low-intensity morning training following carbohydrate restriction overnight) or a control that consumed the same daily carbohydrate (6 g/kg/day) but with normal-to-high carbohydrate availability throughout training sessions. Overall, the sleep-low group experienced greater improvements in cycling-to-exhaustion performance at 150% peak aerobic power (+ 12.5 ± 19.0% vs + 1.63 ± 12.4%; *p* = 0.06), 10-km running time (− 2.9 ± 2.2% vs − 0.1 ± 2.0%, *p* < 0.05), and relative fat mass reduction (− 8.5 ± 7.4% vs − 2.6 ± 7.4%; *p* < 0.01) after three weeks.

The translation of this literature to the unique demands and circumstances of professional cycling poses a challenge. On one hand, over-fueling with carbohydrate on the bike, especially during low- to moderate-intensity training sessions, could blunt metabolic, performance, and body composition adaptations over time. As such, other authors have recommended a “fuel for the work required” approach, which in essence means that, “[carbohydrate] availability is adjusted in accordance with the demands of the upcoming training session(s)” [101]. On the other hand, the practice of applying high-carbohydrate fueling more regularly in training could, at least in theory, allow athletes to sustain higher workloads and remain better motivated over time, which may ultimately lead to improved performance [102]. In the context of a Grand Tour, the main priority for most cyclists is to recover as quickly and fully as possible between stages so that they can optimize performance. In other words, optimizing long-term training adaptations is often not their main focus during the event.

## Personalizing Carbohydrate Fueling Prescriptions

Research to date has observed some degree of inter-individual variability in the ability to oxidize exogenous carbohydrate [4, 5, 103]. The factors explaining this between-person variability are still being explored but likely include body anthropometrics, absolute work rate, environmental conditions, training status, experience with ingesting carbohydrate during exercise (e.g., carbohydrate-focused gut training), and co-ingestion of other nutrients like caffeine [96, 103–106].

At least two investigations have found that body attributes (mass, height, surface area) positively correlate with exogenous carbohydrate oxidation rates [103, 104]. Correlation sizes between body mass and peak exogenous oxidation rates had a range of *r* = 0.46–0.61 in these studies, with similar effect sizes for height (*r* = 0.59) and body surface area (*r* = 0.67). Podlogar et al. [103] found that absolute power output during exercise (at 95% of the first lactate threshold) explained 36% of the variability in peak exogenous glucose oxidation. They also found that 68% of the variability in exogenous glucose oxidation could be explained through the combination of height and power output in multiple regression modeling. Given that both studies had small sample sizes (*n* < 30) [103, 104], additional research verifying these relationships is warranted.

Acute nutritional choices including ingestion of energy, carbohydrate, and various supplements can also impact the capacity to oxidize ingested carbohydrate during exercise [105, 106]. Yeo et al. [106] investigated acute caffeine ingestion and found that a dose of 5 mg/kg of body mass per hour increased exogenous glucose oxidation as well as total carbohydrate oxidation during moderate-intensity cycling. Hearris et al. [4] fed trained cyclists 120 g/h of carbohydrate from various sources (fluid, semisolid gels, solid jelly chews, or a combination) during exercise and reported no significant differences in peak exogenous oxidation rates or oxidation efficiency. This indicates that when carbohydrates are ingested at very high rates, delivery form is unlikely to have a large impact on exogenous oxidation rates, at least under the conditions of the exercise task studied (180 min at 95% lactate threshold with relatively low thermal stress).

Beyond acute interventions, Cox et al. [96] reported that chronically supplementing the diet with carbohydrates (1.5 g/kg body weight for every hour of exercise performed daily) for a 28-day period increased exogenous glucose oxidation during exercise by ~ 16%, which represented a larger increase than from a diet supplemented with protein and fat. A proposed mechanism to explain this enhancement was an up-regulation of intestinal glucose absorption, although no direct measurements were taken to confirm this suggestion. Nonetheless, other lines of research imply that multiple aspects of gastrointestinal function are trainable with repeated exposure to specific nutrients [89, 107].

Due to the inter-person variability in exogenous carbohydrate oxidation, personalizing during-exercise carbohydrate prescriptions based on laboratory testing has become a growing strategy of interest. In a self-described proof-of-concept study, Podlogar et al. [103] utilized beverages enriched with U-^13^C to estimate glucose oxidation rates during 150-min cycling exercise trials carried out at 95% of first lactate threshold. Glucose was provided at 90 g/h during the first exercise trial, and the associated peak exogenous glucose oxidation was used to develop a personalized glucose dose for a second trial (the obtained peak rate was assumed to represent 80% of the dose for the individualized trial). The personalized dose provided 28 ± 11% less glucose (mean: 65 ± 10 vs 90 g/h) without differences in peak exogenous glucose oxidation (personalized: 0.91 ± 0.19 g/min; 90 g/h: 0.90 ± 0.15 g/min; *p* = 0.977). In addition, stomach fullness was modestly lower in the personalized trial.

While the protocol described in Podlogar et al. [103] provides a starting point for research into the practice of personalizing carbohydrate intakes, there are some important caveats worth mentioning. First, the study used glucose-only feedings, meaning that translation to modern fueling where a combination of maltodextrin/glucose–fructose is typically used is less certain. Second, identifying an optimal, personalized carbohydrate intake should perhaps involve multiple trials (at least three) of varying feeding rates so that an individualized oxidation curve can be created. Gastrointestinal symptomology should also be more comprehensively evaluated, as it is an important determinant of whether carbohydrate feeding has positive effects on performance, regardless of how much ingested carbohydrate is oxidized [91].

From a broader perspective, the widespread application of this type of individualized testing is perhaps somewhat limited in practice due to the resources, equipment, and expertise needed as well as the associated costs. Nonetheless, increased adoption by some cycling teams (at least well-resourced ones) is likely, even though questions remain about its benefits given a lack of data on a variety of outcomes, most notably performance. Furthermore, additional research on the day-to-day reliability and minimal detectable change of exogenous carbohydrate oxidation assessments is needed. Without this information, it is difficult to know whether differences observed between repeat tests represent true change or measurement noise.

## Conclusions and Future Directions

**Fig. 6:**
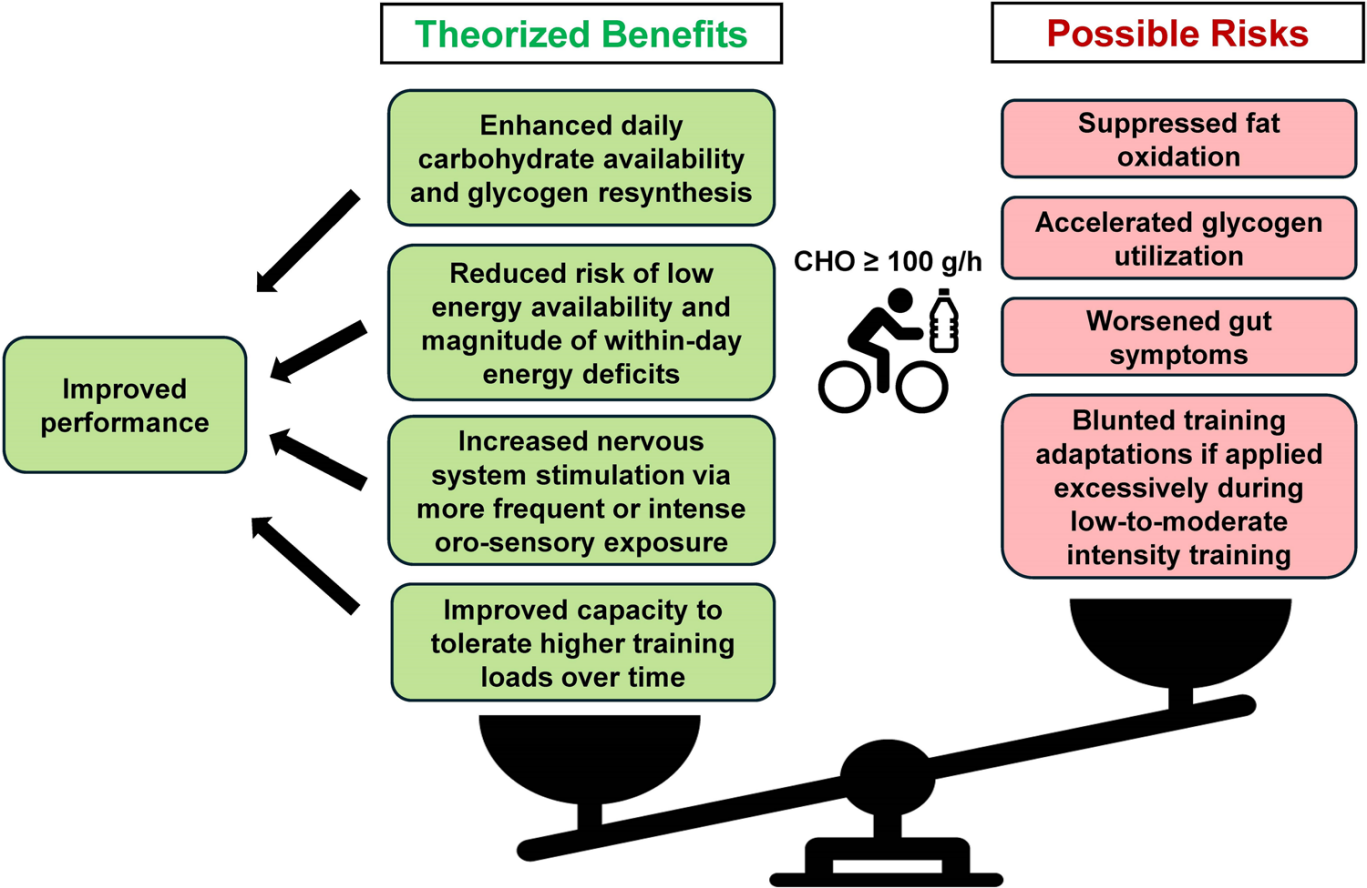
A conceptual overview of the possible benefits and risks of high-carbohydrate fueling. The benefit–risk ratio may be more favorable in the context of a multi-week stage race like a Grand Tour as compared with a 1-day race or during regular background training. However, due to mixed findings and the indirect nature of available evidence, individual athletes and practitioners should use their judgement and experience to make informed decisions about fueling strategies. *CHO* carbohydrate

Anecdotal accounts and some observational studies tentatively support the idea that on-bike carbohydrate fueling has increased over time in the professional peloton. Available research does not directly support performance-enhancing effects of ingesting carbohydrate at ≥ 100 g/h relative to 60–90 g/h. However, studies underpinning this conclusion seemingly did not familiarize participants to such high intakes, and the exercise testing protocols used have not necessarily reflected the conditions and demands that cyclists face when they participate in multi-week stage races or when undertaking intensified training. These situations are defined by extremely high cumulative energy and carbohydrate demands, along with a need to recover quickly each day. Indirect sources of evidence suggest that high-carbohydrate fueling could contribute to improved performance via several mechanisms, mainly by enhancing daily carbohydrate availability, reducing low energy availability, reducing within-day energy deficits, and stimulating the nervous system via oral exposure. Still, cyclists and practitioners need to weigh these potential benefits against possible risks such as suppressed fat oxidation, accelerated glycogen use, increased gastrointestinal symptoms, and reduced responses to chronic training (if applied overzealously). A conceptual overview of these issues is presented in Fig. 6. Given the largely mixed and inconclusive evidence to date, it is impractical to make definitive statements about these tradeoffs, which means that individual athletes and practitioners need to use judgement and experience when making decisions about fueling strategies.

Numerous gaps exist in the literature on high-carbohydrate fueling. Experimental studies examining performance effects should be a priority, keeping in mind the conditions under which professional cyclists often compete. Moreover, whether participants are already accustomed to high-carbohydrate fueling should also be reported in these future trials. Additional observational work is needed to evaluate the prevalence of high-carbohydrate fueling in the professional peloton and whether factors such as body size, rider role in the team, sex, and time of year have any impact. More research is clearly needed to examine whether on-bike high-carbohydrate fueling improves day-to-day energy availability and reduces within-day energy deficits and how any associated changes impact various biomarkers (hormonal, immune, bone), health, and performance. Future research should also explore how personalization of fueling based on exogenous carbohydrate testing impacts gastrointestinal symptoms, metabolic outcomes, and performance. High-carbohydrate fueling appears to be firmly entrenched in the peloton, and addressing these and other questions will bring additional scientific data to a practice that has largely been driven by anecdotes and speculation.

## Declarations

### Availability of data and material

Underlying data for the correlational analyses presented in the article are available within the cited sources.
